# CRISPR/Cas13a-based rapid detection method for porcine deltacoronavirus

**DOI:** 10.3389/fmicb.2024.1429486

**Published:** 2024-07-25

**Authors:** Ran Luo, Zhimeng Cheng, Haoyu Wang, Qiyue Yang, Yongping Zeng, Yijun Yang, Yuankun Chen, Wenting Li, Xiao Liu

**Affiliations:** ^1^College of Veterinary Medicine, Southwest University, Chongqing, China; ^2^Division of Biliary Tract Surgery, Department of General Surgery, West China Hospital, Sichuan University, Chengdu, Sichuan, China; ^3^Research Center for Biliary Diseases, West China Hospital, Sichuan University, Chengdu, Sichuan, China; ^4^Department of Infectious and Tropical Diseases, The Second Affiliated Hospital of Hainan Medical University, Haikou, China; ^5^Department of Infectious Diseases, The First Affiliated Hospital of Anhui Medical University, Hefei, China; ^6^State Key Laboratory of Silkworm Genome Biology, Chongqing, China

**Keywords:** CRISPR/Cas13a, PDCoV, detection method, rapid detection, RPA

## Abstract

**Background:**

Porcine deltacoronavirus (PDCoV) is a newly discovered porcine intestinal pathogenic coronavirus with a single-stranded positive-sense RNA genome and an envelope. PDCoV infects pigs of different ages and causes acute diarrhea and vomiting in newborn piglets. In severe cases, infection leads to dehydration, exhaustion, and death in sick piglets, entailing great economic losses on pig farms. The clinical symptoms of PDCoV infection are very similar to those of other porcine enteroviruses. Although it is difficult to distinguish these viral infections without testing, monitoring PDCoV is very important because it can spread in populations. The most commonly used methods for the detection of PDCoV is qPCR, which is time-consuming and require skilled personnel and equipment. Many farms cannot meet the conditions required for detection. Therefore, it is necessary to establish a faster and more convenient method for detecting PDCoV.

**Aims:**

To establish a rapid and convenient detection method for PDCoV by combining RPA (Recombinase Polymerase Isothermal Amplification) with CRISPR/Cas13a.

**Methods:**

Specific RPA primers and crRNA for PDCoV were designed, and the nucleic acids in the samples were amplified with RPA. Fluorescent CRISPR/Cas13a detection was performed. We evaluated the sensitivity and specificity of the RPA–CRISPR/Cas13a assay using qPCR as the control method.

**Results:**

CRISPR/Cas13a-assisted detection was completed within 90 min. The minimum detection limit of PDCoV was 5.7 × 10^1^ copies/μL. A specificity analysis showed that the assay did not cross-react with three other porcine enteroviruses.

**Conclusion:**

The RPA–CRISPR/Cas13a method has the advantages of high sensitivity, strong specificity, fast response, and readily accessible results, and can be used for the detection of PDCoV.

## 1 Introduction

Porcine deltacoronavirus (PDCoV) is a new type of porcine intestinal coronavirus discovered in the past decade. At the end of 2012, Woo et al. detected deltacoronavirus in pigs for the first time in Hong Kong, China, and designated it PDCoV HKU-15 (Woo et al., 2012). The incidence of swine infection was first reported in Ohio, USA (Wang et al., 2014), and the disease has since been reported in many countries in the Americas and Asia, including Canada (Pasick et al., 2014), Mexico (More-Bayona et al., 2022), Thailand (Pérez-Rivera et al., 2019), Vietnam (Janetanakit et al., 2016), South Korea (Lorsirigool et al., 2016), Japan (Lee and Lee, 2014), and China (Zhang et al., 2019). PDCoV, porcine transmissible gastroenteritis virus (TGEV), and porcine epidemic diarrhea virus (PEDV) are common enteroviruses in pigs. The symptoms and pathogenesis of these viruses are similar, and mixed infections occur. It is difficult to differentiate these viruses without laboratory diagnoses (Wang et al., 2019). It is noteworthy that PDCoV strains have been isolated from plasma samples from children with acute fever, indicating that PDCoV is highly likely to be transmitted in human populations (Lednicky et al., 2021). Furthermore, it has been demonstrated that PDCoV can infect human cell lines by interacting with aminopeptidase N (APN) (Li et al., 2018). This emphasizes the risk of the cross-species transmission of PDCoV, which may even spread among people, so it is important to strengthen the prevention, control, and monitoring of PDCoV.

Common diagnostic methods for PDCoV include electron microscopy, reverse transcription–PCR, enzyme-linked immunosorbent assay (ELISA) (Zhang, 2016). qPCR is often used as the gold standard detection method on pig farms (Zhao et al., 2023), however, the process is time-consuming and complex, requiring professional personnel and equipment. As a result, it is not suitable for ordinary pig farms or other situations with limited experimental resources. Therefore, a better PDCoV detection method is required.

The clustered regularly interspaced short palindromic repeats (CRISPR)/CRISPR-associated (Cas) system is a novel technology for pathogen detection. The SHERLOCK *in vitro* nucleic acid detection platform established by Gootenberg et al. (2017) uses the CRISPR/Cas technology combined with RPA to detect pathogens. RPA is first used for nucleic acid amplification, and the virus is then transcribed into single-stranded RNA (ssRNA), which is finally detected by the Cas13a protein of *Leptotrichia wadei* (LwCas13a). After the CRISPR RNA (crRNA) recognizes the target sequence, the LwCas13a protein is activated, and the nearby nontarget RNA is subjected to incidental cleavage. The presence of the target RNA is determined by observing whether the quenched fluorescent RNA releases a fluorescent signal (Gootenberg et al., 2017). More recently, a detection platform for double-stranded DNA (dsDNA) using CRISPR/Cas12a (Cpf1 protein) was established (Chen et al., 2018). The CRISPR/Cas nucleic acid detection technology has been used to detect various viruses, including SARS-CoV-2 (Jiao et al., 2021), and can also be combined with lateral chromatography to quickly and easily read the results without equipment (Gootenberg et al., 2018).

Piepenburg et al. (2006) discovered the RPA technology, which allows for exponential amplification of nucleic acids under low temperature and constant temperature conditions. This method is renowned for its sensitivity and rapidity (Piepenburg et al., 2006). At present, RPA technology has been widely employed in the detection of pathogens and can be integrated with other methods to improve the efficiency of detection (Lobato and O’Sullivan, 2018).

Therefore, to establish a faster, more accurate, and more convenient detection method, we developed a new convenient and rapid detection platform for PDCoV by combining RPA with CRISPR/Cas13a. The advantages of this method are its simplicity of operation, its short detection time, the easily read results, and the portable detection of PDCoV with high sensitivity and high specificity.

## 2 Materials and methods

### 2.1 Viruses, clinical samples, and extraction of RNA

The strains of PDCoV, TGEV, Seneca virus A (SVA), Porcine Rotavirus (PoRV), and PEDV used in this study were all from Laboratory 308 of Southwest University College of Veterinary Medicine, Chong Qing, China. The clinical blood samples utilized in our study were sourced from XUKE Bioengineering Co., Ltd., which specializes in providing blood samples from pig farms located in various regions of Sichuan, China. The RNA was extracted from the samples using the phenol–chloroform method. Trizol Reagent (1 mL) was added to each sample in a centrifuge tube, which was shaken violently for 30 s and allowed to stand at room temperature for 1 min. Then 200 μL of chloroform was added to the tube, which was shaken violently for 15 s, allowed to stand at room temperature for 1 min, and centrifuged at 12,000 rpm for 5 min. After centrifugation, 400 μL of supernatant was removed into a new RNase-free centrifuge tube, and 500 μL of isopropanol was added, before violent shaking for 15 s. Each sample was allowed to stand at room temperature for 1 min, and were then centrifuged at 10,000 rpm for 5 min. The supernatant was discarded, 1 mL of ice-cold 75% ethanol was added, and the sample was centrifuged at 7500 rpm for 5 min. After the supernatant was discarded, ventilate in the fume hood (note that RNA cannot be completely dried). Finally, 20 μL of diethyl pyrocarbonate (DEPC)-treated water was added to the centrifuge tube, mixed well, and stored at −80°C.

### 2.2 Synthesis of standard plasmids, primers, crRNA, and reporter RNA

To ensure the specificity of the detection method, the sequences of the plasmids, primers, and crRNA used in this study were highly conserved. The conserved N gene sequence (1,029 bp in total) of PDCoV (GenBank: MK330604.1) was obtained with an alignment analysis in the National Center for Biotechnology Information (NCBI) database. The sequence was inserted into the pUC57 and sent to GenScript Biotechnology Co., Ltd. for the synthesis of pUC57–PDCoV as the standard positive plasmid. Cells were transformed with the synthesized plasmid and the plasmid was then extracted from the cells. The plasmid concentration was determined and the plasmid cryopreserved as the template for the subsequent detection of PDCoV. The conserved sequence of PDCoV was obtained with an alignment analysis in NCBI, and the primers for RPA were designed by NCBI. Three pairs of primers were designed for RPA, and the T7 promoter sequence was added before the 5′ primer to facilitate subsequent *in vitro* transcription. crRNA design reference Zhang Feng laboratory Cas13a-related articles (Kellner et al., 2019), a total of two designs. The crRNA consists of a 36 nt long repeat sequence and a 29 nt long spacer sequence. We designed spacer sequences manually, and used SnapGene 7.0.2 to check secondary structure. The reporterRNA sequence 5′ modified as FAM, 3′ modified as BHQ1. The designed primer, crRNA, and reporter RNA sequences were sent to Shenzhen BGI Co., Ltd. for synthesis. The sequences used in this study are shown in Table 1. Details of crRNA are shown in Table 2.

**Table 1:** Sequences used in this study.

**Name**	**Sequence (5’-3’)**
RPA-F1	**TAATACGACTCACTATAGGG**CAAGGGTAAAACCATTTCTCAGGTATTTGG
RPA-F2	**TAATACGACTCACTATAGGG**CCAAGGGTAAAACCATTTCTCAGGTATTTG
RPA-F3	**TAATACGACTCACTATAGGG**AACCGGTCTCGTACTGGTGCCAATGTCGGCT
RPA-R1	TTTTTAGGTTTCTTCTGCTGTTTGGGTTTA
RPA-R2	CAGAGTTACCTTTTTAGGTTTCTTCTGCTG
RPA-R3	TGATTGAGTACGAGAAGGTAAGGGTAATTG
crRNA1	**GAUUUAGACUACCCCAAAAACGAAGGGGACUAAAA**CAGCGAAAAGCAUUUCCUGAACACCAGGC
crRNA2	**GAUUUAGACUACCCCAAAAACGAAGGGGACUAAAA**ACCCGUCUUCUCAGUGUCUGCAGAGCCGA
reporterRNA	5′/6-FAM/UUUUUU-BHQ1/3’

Primer (T7 promoter sequence was thickened), crRNA (repeat sequence was thickened), and reporter RNA.

**Table 2:** Length of the spacers sequence and location of the spacers sequence in the target gene.

**Name**	**Spacer sequence (29 nt)**	**Target gene**	**Site**
crRNA1	CAGCGAAAAGCAUUUCCUGAACACCAGGC	GCCUGGUGUUCAGGAAAUGCUUUUCGCUG	708–736
crRNA2	ACCCGUCUUCUCAGUGUCUGCAGAGCCGA	UCGGCUCUGCAGACACUGAGAAGACGGGU	641–669

### 2.3 Nucleic acid RPA

The amplification process was performed according to the instructions of the DNA thermostatic rapid amplification kit (Amplification Future Changzhou Biotechnology Co., Ltd). For the reaction system, 29 μL of buffer A was added to each tube, followed by 2 μL of the upstream primer (10 μM) and 2 μL of the downstream primer (10 μM), 5 μL of the standard plasmid template (468 ng/μL), and 9.1 μL of DEPC-treated water. Finally, 2.5 μL of buffer B was added to the reaction tube and fully mixed. Immediately after mixing, the reaction tube was placed in a thermostatic device at 37°C for 30 min. In order to facilitate agarose gel electrophoresis, after the reaction, 50 μL of a solution of Tris-saturated phenol, chloroform, and isoamyl (Woo et al., 2012; Tian et al., 2023; Li et al., 2024) was added to the reaction product. After mixing evenly, the sample was centrifuged at 12,000 rpm for 5 min, and the supernatant was mixed with 6 μL of 6 × loading buffer. The mixed product can be directly used for agarose gel electrophoresis.

### 2.4 Establishment of CRISPR/Cas13a detection system

RNase inhibitor (1 μL), 2 μL of LwCas13a (GenScript Biotechnology Co., Ltd), 2 μL of crRNA (7.5 ng/μL), 1.2 μL of NTP mix (7.5 mM), 0.4 μL or T7 RNA polymerase, 0.3 μL of 10 × T7 reaction buffer, 5 μL of FAM-BHQ1 RNA reporter (6 pmol/μL), 5 μL of 10 × Cas13a reaction buffer, 2 μL of RPA product, and 36.1 μL of RNase-free water were added to a 200 μL PCR tube. The reaction was performed at 37°C for 40 min, the fluorescent signal was collected every 30 s on Bioer Line Gene 9600 Plus Real Time Thermalcycler (FQD-96A, Hangzhou Bori Technology Co., Ltd).

### 2.5 Optimization of reaction conditions

We optimized the conditions of the whole reaction process, including the reaction time (10, 20, 30, and 40 min), reaction temperature (35, 36, 37, 38, 39, and 40°C), Cas13a protein concentration (50, 100, and 150 ng/μL), and reporter RNA concentration (10, 8, 6, 4, and 2 pmol/μL), by monitoring the fluorescence emitted by the CRISPR/Cas13a detection system.

### 2.6 Evaluating detection capability

Evaluation of specificity: Strains of PDCoV, TGEV, SVA, PEDV and PoRV were amplified using the RNA thermostatic rapid amplification kit (Amplification Future Changzhou Biotechnology Co., Ltd). For the reaction system, 29 μL of buffer A was added to each tube, followed by 2 μL of the upstream primer (10 μM) and 2 μL of the downstream primer (10 μM), 5 μL of the Virus samples, and 9.5 μL of DEPC-treated water. Finally, 2.5 μL of buffer B was added to the reaction tube and fully mixed. Immediately after mixing, the reaction tube was placed in a thermostatic device at 42°C for 30 min. The products were added to the established CRISPR/Cas13a detection system. The reaction was performed at 37°C for 40 min, and the fluorescent signal was collected every 30 s with the Bioer Line Gene 9600 Plus Real Time Thermalcycler.

Evaluation of sensitivity: The PDCoV plasmid standard was diluted to 5.7 × 10^7^, 5.7 × 10^6^, 5.7 × 10^5^, 5.7 × 10^4^, 5.7 × 10^3^, 5.7 × 10^2^, 5.7 × 10^1^, and 5.7 × 10^0^ copies/μL, and amplified with RPA for 30 min. Then the RPA reaction product was added 2 μL to the CRISPR/Cas13a detection system at 37°C for 40 min, and the fluorescent signal was collected every 30 s on the Bioer Line Gene 9600 Plus Real Time Thermalcycler. We simultaneously used the same concentrations of the standard plasmid for an qPCR analysis and compared the results.

### 2.7 Evaluating detection repeatability

We used high (5.7 × 10^6^ copies/μL), medium (5.7 × 10^3^ copies/μL), and low (5.7 × 10^1^ copies/μL) concentrations of the PDCoV plasmid standard. After RPA amplification for different times, we used the CRISPR/Cas13a system to detect and evaluate the repeatability of detection.

### 2.8 Evaluating sample detection

In this experiment, we used both the CRISPR/Cas13a method and qPCR to detect PDCoV in the same samples, and compared and analyzed the results of the two methods.

## 3 Results

### 3.1 Construction of CRISPR/Cas13a detection system

**Figure 1:**
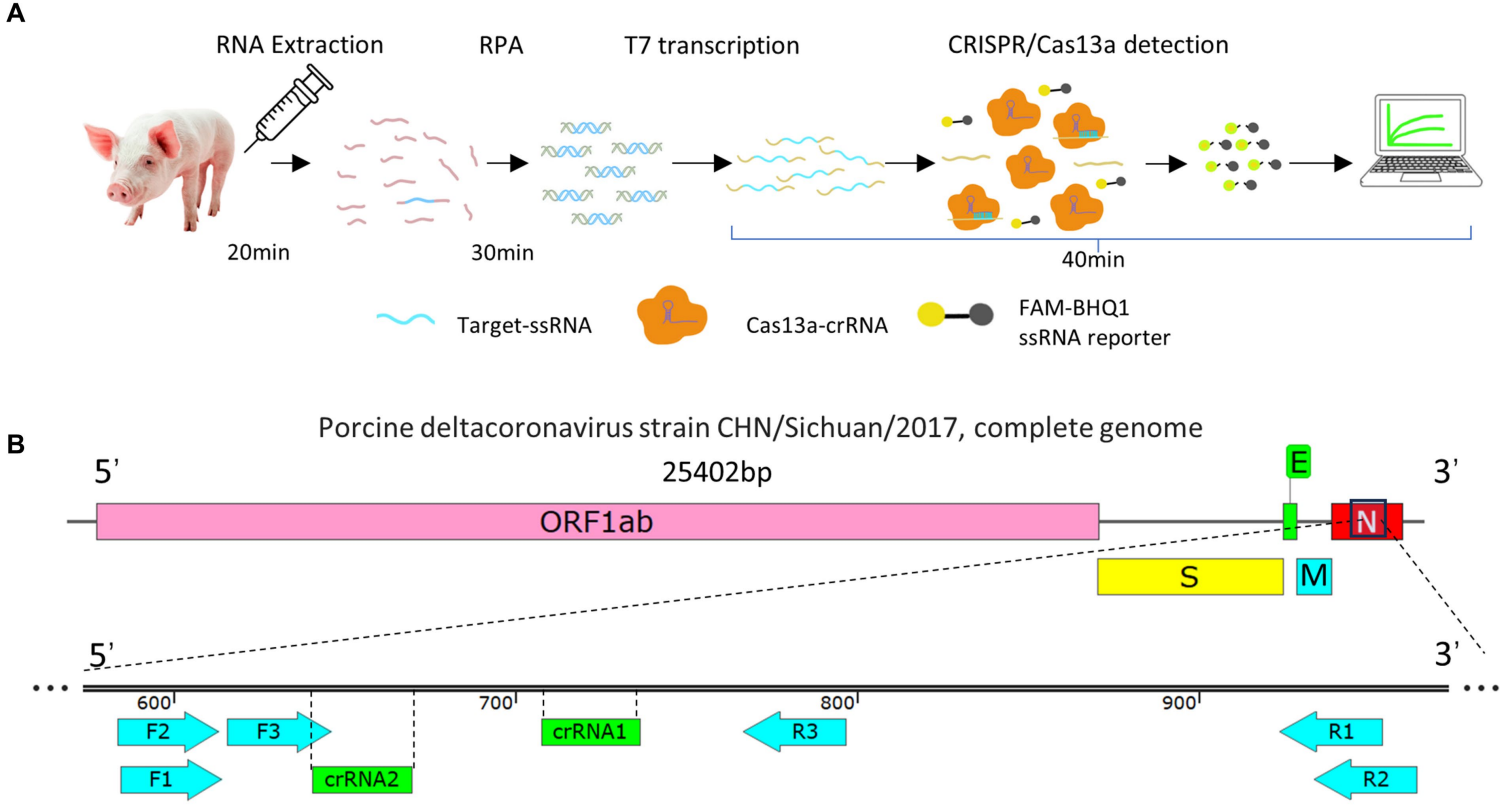
**(A)** Flow diagram of the RPA–CRISPR/Cas13a detection method. **(B)** Schematic representation of the design of the RPA primers and crRNA. Three pairs of RPA primers and two crRNAs were designed based on the conserved fragment of the N region of PDCoV.

The RPA–CRISPR/Cas13a detection system steps are as follows. After RNA was extracted from the samples, it was processed with reverse transcription and RPA amplification, and the RPA product was then added to the CRISPR/Cas13a system. The T7 in the system was reverse transcribed into ssRNA, which was recognized by crRNA and activated the bystander activity of the Cas13a protein, so that the reporter RNA was cut and, consequently, emitted a fluorescent signal. We directly observed this fluorescence under blue and ultraviolet irradiation, or used instruments to quantify the fluorescent signal (Figure 1A).

**Figure 2:**
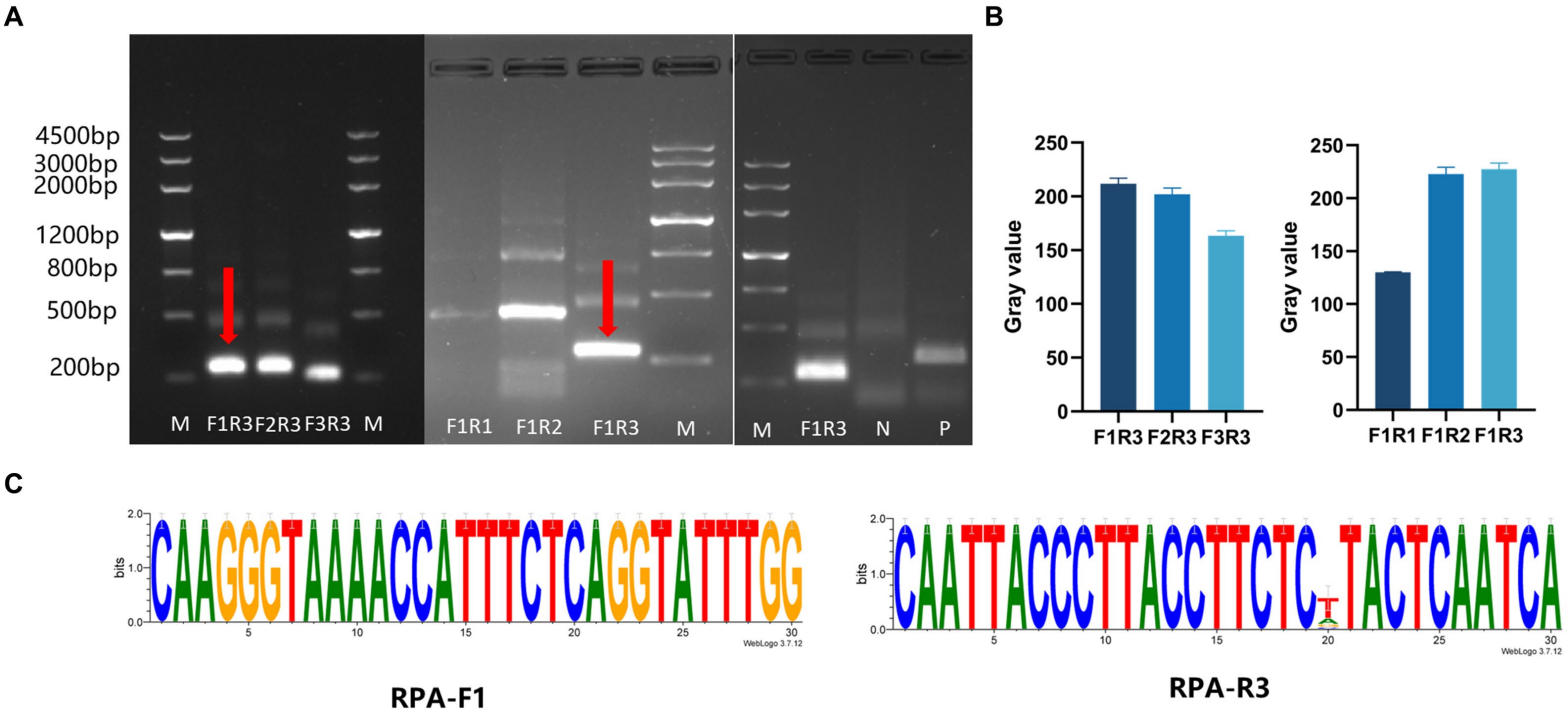
Screening the RPA primers and crRNAs. **(A)** Screening the RPA primers. First, the downstream primer R3 was selected to screen the upstream primers (F1, F2, and F3), and F1 showed the highest efficiency. Then F1 was used to screen the downstream primers (R1, R2, and R3). The upstream primer F1 and the downstream primer R3 were the best primer combination. **(B)** ImageJ was used to detect the gray value of the image, which was repeated three times, and draws a histogram. **(C)** Conservative analysis of RPA primers.

**Figure 3:**
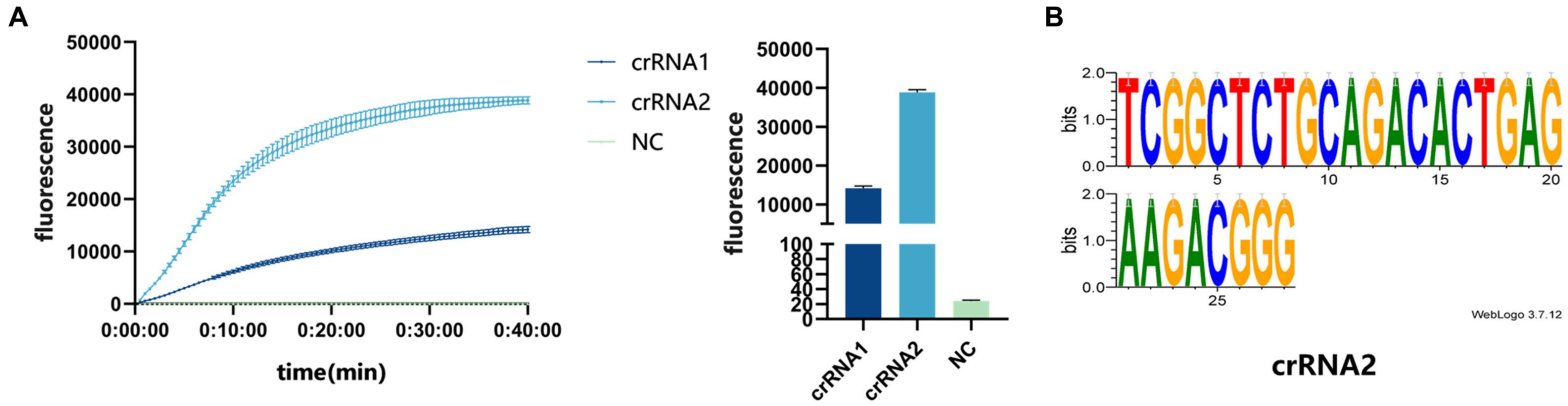
**(A)** crRNA screening. The real-time fluorescence curve and the end-point fluorescence value diagram indicate that crRNA2 had the better effect. **(B)** Conservative analysis of crRNA2.

Three pairs of RPA primers and two crRNAs were designed based on the conserved fragment of the N region of PDCoV (Figure 1B). The constructed PDCoV standard plasmid was RPA amplified with three sets of primers. Agarose gel electrophoresis showed that primer pair F1R3 achieved the best amplification efficiency and it was used in subsequent experiments (Figures 2A,B). The CRISPR/Cas13a system was used to detect the fluorescent signal, and the results showed that crRNA2 had the best cleavage efficiency (Figure 3A). A WebLogo^1^ analysis confirmed the good conservation of the F1R3 primer pair (Figure 2C) and crRNA2 (Figure 3B). Therefore, primers F1R3 and crRNA2 were used to establish an effective detection system.

### 3.2 Optimized reaction conditions

**Figure 4:**
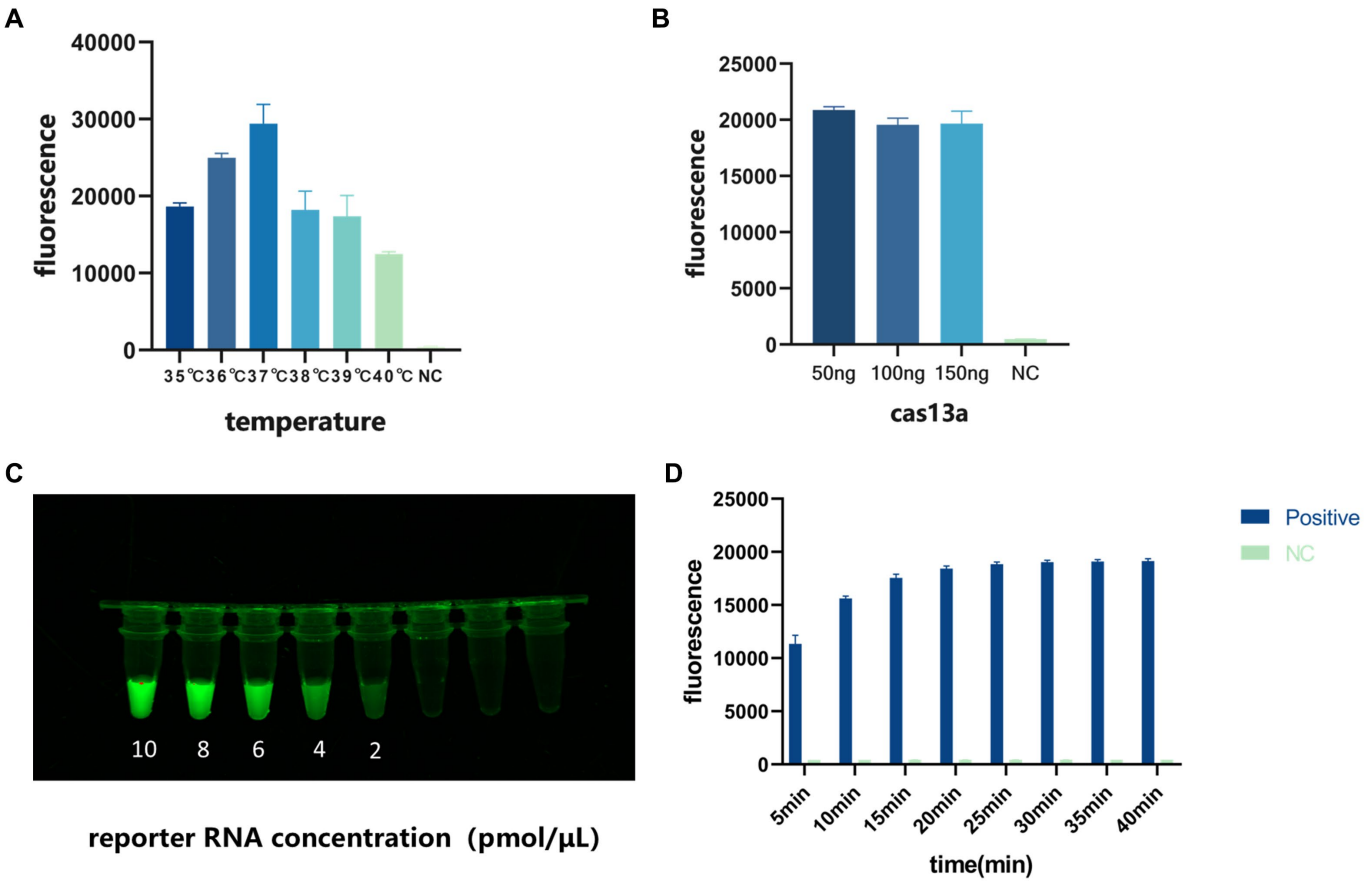
Optimization of the conditions of the detection system. **(A)** Optimization of reaction temperature. **(B)** Optimization of Cas13a protein concentration. **(C)** Optimization of reporter RNA concentration. **(D)** Optimization of reaction time.

We used the fluorescent signal emitted by the CRISPR/Cas13a system to optimize the temperature of the entire reaction, the Cas13a protein concentration, and the reporter RNA concentration. The optimization process examined single variables, including the reaction time (10, 20, 30, and 40 min), reaction temperature (35, 36, 37, 38, 39, and 40°C), the Cas13a protein concentration (50, 100, and 150 ng/μL), and the reporter RNA concentration (10, 8, 6, 4, and 2 pmol/μL). The results showed that the reaction efficiency was highest at 37°C (Figure 4A), that 50 ng/μL Cas13a protein was very highly reactive (Figure 4B), and that the fluorescent signal could be seen with the naked eye at an RNA reporter concentration of 2 pmol/μL (Figure 4C). When the reaction was conducted for 40 min, the fluorescence value reached its peak and subsequently stabilized. Therefore, we have determined that 40 min is the optimal reaction time. (Figure 4D).

### 3.3 Detection capacity of CRISPR/Cas13a system

**Figure 5:**
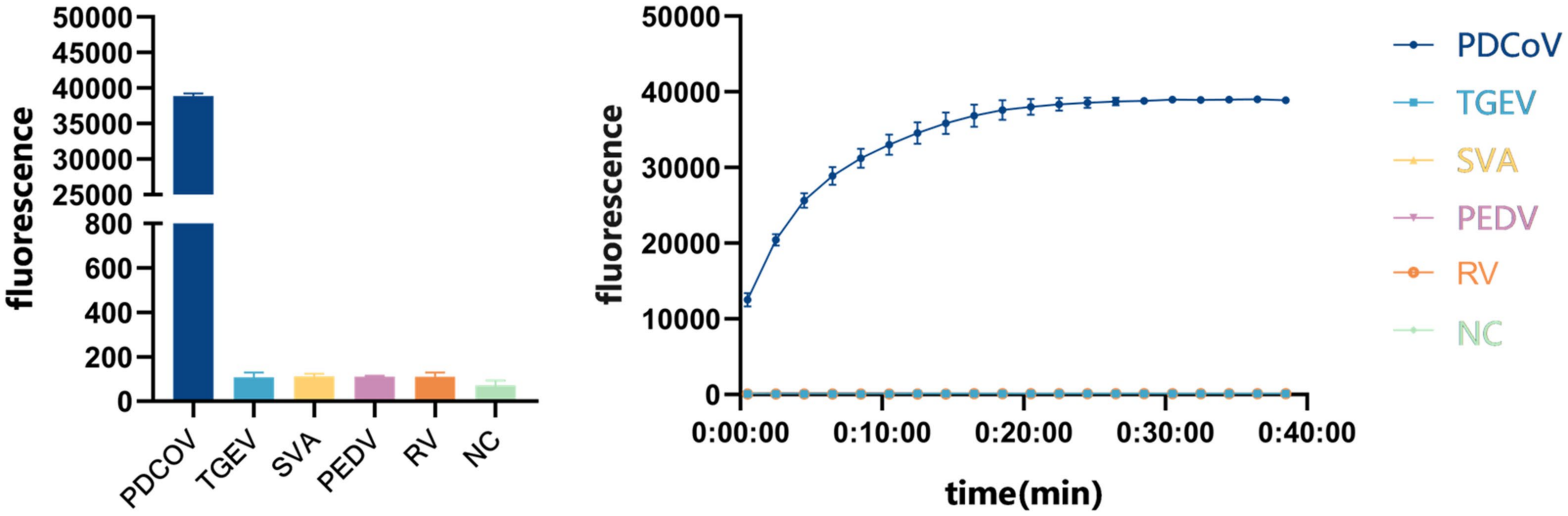
Specificity evaluation of CRISPR/Cas13a system to detect PDCoV.

We evaluated the detection capacity of the CRISPR/Cas13a system by assessing its sensitivity and specificity. To evaluate its specificity, strains of PDCoV, TGEV, SVA, PEDV and PoRV were added to the established CRISPR/Cas13a system, and RNase-free water was used as the template in the negative control. Only the PDCoV sample tested positive, and the other viral samples tested negative, indicating that the system has good specificity (Figure 5).

**Figure 6:**
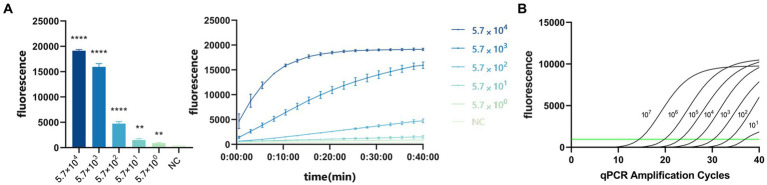
**(A)** Sensitivity of CRISPR/Cas13a system to detect PDCoV. When the fluorescence value was higher than 893.86, the sample was judged to be positive. ****p < 0.0001, **p < 0.01. **(B)** Sensitivity of RT–qPCR. When the Ct value is less than 35, the sample is judged to be positive.

To evaluate the sensitivity of the system, we diluted the standard PDCoV plasmid to 5.7 × 10^7^, 5.7 × 10^6^, 5.7 × 10^5^, 5.7 × 10^4^, 5.7 × 10^3^, 5.7 × 10^2^, 5.7 × 10^1^, and 5.7 × 10^0^ copies/μL for RPA amplification. The reaction product was added to the CRISPR/Cas13a system at 37°C for 40 min, and the fluorescent signal was collected every 30 s on the Gene-9600 Thermalcycler. According to the final fluorescence value, when the fluorescence value was higher than 893.86, the sample was judged to be positive. At the same time, qPCR was used to analyze the same concentrations of the template. When the Ct value is less than 35, the sample is judged to be positive. The results showed that the CRISPR/Cas13a system detected as little as 10^1^ copies of the PDCoV plasmid (Figure 6A), as did qPCR (Figure 6B).

### 3.4 Repeatability

We used high (5.7 × 10^6^ copies/μL), medium (5.7 × 10^3^ copies/μL), and low (5.7 × 10^1^ copies/μL) concentrations of the PDCoV standard to perform RPA–CRISPR/Cas13a detection at different times to evaluate the repeatability of detection. The experimental results showed that the high, medium, and low concentrations of the PDCoV standard were detected at three different time points, confirming that the detection system has good repeatability (Table 3).

**Table 3:** The repeatability assessment of CRISPR/Cas13 system.

	5.7 × 10^6^ copies/μL	5.7 × 10^3^ copies/μL	5.7 × 10^1^ copies/μL
Coefficient of variation	1.609%	1.155%	7.177%

The coefficient of variation was calculated using Graph Pad prism8.0.2 software.

### 3.5 Sample detection

We collected 123 samples to verify the reliability of the CRISPR/cas13a system in detecting clinical PDCoV samples. The extracted samples were detected with both the qPCR and RPA–CRISPR/Cas13a methods. As shown in Table 4, 39 samples were positive and 84 samples were negative with qPCR, whereas 36 samples were positive and 87 samples were negative with the CRISPR/Cas13a method. Therefore, the results of the two methods were consistent (*k* = 0.94).

**Table 4:** Sensitivity, specificity, and kappa value of CRISPR/Cas13 and qPCR were determined.

		**qPCR**		**total**	**sensitivity**	**specificity**	**Kappa value**
		+	−				
**CRISPR/Cas13a**	+	36	0	36	92.3%	100%	0.94
	−	3	84	87			
	Total	39	84	123			

## 4 Discussion

The CRISPR/Cas system exists widely in prokaryotes and is an acquired immune system. It recognizes foreign viral gene fragments and cuts them into itself. After recognizing the corresponding pathogens, it produces a corresponding crRNA, which guides the Cas protein to recognize and degrade specific target sequences to protect the prokaryote from viral invasion (Pausch et al., 2020). The Cas13a (C2c2) protein, like Cas9 and Cas12, has incidental cleavage activity. After recognizing the specific target under the guidance of crRNA, its activated nuclease activity cleaves nonspecific nucleic acid fragments (East-Seletsky et al., 2017). To date, CRISPR/Cas13a has been used to detect a variety of pathogens, including hepatitis E virus (Li et al., 2024), hepatitis B virus (Tian et al., 2023), avian influenza virus (Li et al., 2023), *Vibrio alginolyticus* (Wang et al., 2023), and Trichomonas vaginalis (Yang et al., 2024). However, as far as we know, this technique has not been used to detect PDCoV.

In summary, we have established a new RPA–CRISPR/Cas13a method of PDCoV detection. We designed three pairs of primers based on the N region of PDCoV, and then designed two crRNAs based on the RPA product fragment. After screening, we showed that the F1R3 primer combination and crRNA2 were optimal in this system. We then optimized the reaction conditions, and identified the best reaction temperature as 37°C, the optimal concentration of reporter RNA as 6 pmol/μL, and the optimal concentration of Cas13a protein as 50 ng/μL for the rapid and accurate on-site detection of PDCoV. This detection method is highly specific and does not cross-react with TGEV, SVA, or PEDV. The system is also highly sensitive and can detect copy numbers as low as 101, identical to the qPCR method. Compared with qPCR, the kappa value was 0.94 (k > 0.75), so the results of the two methods are highly consistent. Although RT–qPCR is more accurate in sample detection, the RPA–CRISPR/Cas13a detection method is more time efficient and less labor intensive, and reduced dependence on the instrument, so it is suitable for use on ordinary farms.

The PDCoV detection method established in this study has many obvious advantages (Woo et al., 2012). The combination of the RPA and CRISPR/Cas13a technologies rapidly and efficiently detects PDCoV (Wang et al., 2014). The system has high specificity and sensitivity, and its detection ability is consistent with that of qPCR (Pasick et al., 2014). With the advantages of high sensitivity and high specificity, it is more convenient and less time-consuming than qPCR, so it is a simple and convenient detection method.

Although the method we established has many advantages, there is still room for improvement. We used the phenol–chloroform method to extract the viral RNA. Although the extraction efficiency was high, there were several shortcomings, such as cumbersome and slow processes and the repeated opening of the lid of the reaction vessel, which increased the risk of contamination. Commercial RNA extraction kits are widely used in the industry, and they can serve as an alternative to phenol chloroform for RNA extraction in conventional pig farms. In future studies, we hope to find a more convenient and efficient method for extracting viral RNA to complement the detection system.

PDCoV, as an RNA virus, requires a reverse transcription step before RPA. To improve the convenience of the detection method, we will try to combine the reverse transcription system with the isothermal amplification method in future research. Moreover, RPA and CRISPR/Cas13a are performed in two separate steps. In future studies, we will try to combine the two steps and optimize them to further reduce the reaction time and the contamination risk.

## Data availability statement

Publicly available datasets were analyzed in this study. This data can be found here: https://www.ncbi.nlm.nih.gov/nuccore/MK330604.1.

## Ethics statement

The animal study was approved by Laboratory Animal Center, Southwest University. The study was conducted in accordance with the local legislation and institutional requirements.

## Author contributions

RL: Data curation, Formal analysis, Writing – original draft, Software, Methodology. ZC: Writing – original draft. HW: Writing – original draft, Data curation. QY: Writing – original draft. YZ: Writing – original draft. YY: Resources, Writing – original draft. WL: Resources, Writing – original draft. YC: Resources, Writing – original draft. XL: Funding acquisition, Writing – review & editing.

## Footnotes

^1^ https://weblogo.threeplusone.com
